# Transcatheter mitral valve implantation for degenerated mitral bioprostheses or failed surgical annuloplasty rings: A systematic review and meta‐analysis

**DOI:** 10.1111/jocs.13767

**Published:** 2018-07-10

**Authors:** Junjie Hu, Yan Chen, Sijin Cheng, San Zhang, Kaiqin Wu, Wenli Wang, Yongxin Zhou

**Affiliations:** ^1^ Department of Thoracic‐Cardiovascular Surgery Tongji Hospital Tongji University School of Medicine Shanghai China

**Keywords:** mitral regurgitation, mitral stenosis, TMVIR, TMVIV, transapical, transseptal

## Abstract

**Background:**

Transcatheter mitral valve‐in‐valve (TMVIV) and valve‐in‐ring (TMVIR) implantation for degenerated mitral bioprostheses and failed annuloplasty rings have recently emerged as treatment options for patients deemed unsuitable for repeat surgery.

**Methods:**

A systematic literature review was conducted to summarize the data regarding the baseline characteristics and clinical outcomes of patients undergoing TMVIV and TMVIR procedures.

**Results:**

A total of 245 patients (172 patients who underwent TMVIV surgery and 73 patients who underwent TMVIR surgery) were included in the study; 93.5% of patients experienced successful TMVIV or TMVIR implantation. The mortality rates at discharge, 30 days, and 6 months were 5.7%, 8.1%, and 23.4%, respectively. The transapical (TA) access route was used in most procedures (55.2%). The TA and transseptal (TS) access routes resulted in similar outcomes. No significant differences were observed in the short‐term outcomes between the patients who developed mitral stenosis versus mitral regurgitation as the mode of failure.

**Conclusions:**

TMVIV and TMVIR implantation for degenerated mitral bioprostheses and failed annuloplasty rings are safe and effective. Both procedures, via TA or TS access, can result in excellent short‐term clinical outcomes in patients with mitral stenosis or regurgitation, but long‐term follow‐up data are currently lacking to determine the durability of these procedures.

## 1 INTRODUCTION

Surgical mitral valve repair or replacement remains the gold standard for treating severe symptomatic mitral valve disease. Up to 35% of patients require a repeat operation during the first 10 years, and the in‐hospital mortality rate may be as high as 12%.^1^, ^2^ Furthermore, some patients requiring mitral valve repair/replacement are deemed to be too high risk for repeat surgery. Recently, transcatheter mitral valve interventions have emerged as alternatives to conventional surgical valve replacement in patients requiring repeat surgery. Cheung et al^3^ reported the first transcatheter mitral valve‐in‐valve (TMVIV) implantation in humans in 2009, and the first transcatheter mitral valve‐in‐ring (TMVIR) implantation in humans was performed by de Weger et al in 2011.^4^ Since that time, additional patients have received TMVIV or TMVIR surgery.^5^, ^6^ This study reviews the outcomes of TMVIV implantation for degenerated mitral bioprostheses and TMVIR implantation for failed annuloplasty rings, according to the Mitral Valve Academic Research Consortium (MVARC) criteria.^7^ The results are stratified according to the mitral valve failure mode and the access route. This information may aid in clinical decision making in patients with degenerated mitral bioprostheses or failed annuloplasty rings who are not candidates for repeat surgery.

## 2 METHODS

### 2.1 Search strategies

A comprehensive, systematic search was performed to identify all relevant articles published in the PubMed and Web of Science databases from 2000 to March 30, 2018. The following search terms were used: “transcatheter mitral valve implantation” or “transcatheter mitral valve replacement” or “TMVI” or “TMVR.” This analysis was performed in accordance with the Preferred Reporting Items for Systematic Reviews and Meta‐Analyses (PRISMA) Statement.^8^

### 2.2 Study selection

The inclusion criteria were as follows: (1) patients received either a TMVIV or TMVIR implantation and (2) reported data necessary to assess the baseline characteristics and outcomes. Articles were excluded if any of the following criteria applied: (1) non‐English article; (2) animal experiments; (3) no relevant information on TMVI implantation; (4) lack of details regarding postoperative outcomes; (4) TMVIV or TMVIR for native mitral valve; (5) insertion of a TMVIV or TMVIR during a full sternotomy under direct vision; and (6) meeting abstracts.

### 2.3 Data extraction

The following information was extracted from each study: age, gender, logistic EuroSCORE, the Society of Thoracic Surgeons (STS) score, comorbidities, function of the other valves, history of heart surgery, New York Heart Association (NYHA) class, left ventricular ejection fraction, mitral regurgitation (MR) severity, mean transmitral gradient, prior mitral bioprostheses, death, valve migration, access site, and vascular and other postprocedure complications. For those patients who were reported in two or more articles, we removed the duplicates by checking their age, gender, logistic EuroSCORE or STS score, prior mitral bioprostheses, and the author's contact address. For the subgroup analysis, we recorded the mitral valve failure mode, access route, and size of the transcatheter valve. Two reviewers extracted the data independently using a predefined Excel form.

### 2.4 Statistical analysis

Continuous variables are described as means and standard deviations for normally distributed data, or medians and interquartile ranges for non‐normally distributed data. Differences between continuous variables were analyzed using a *t*‐test. Categorical variables are described with absolute and relative frequencies. Differences between categorical variables were evaluated using the chi‐square test or Fisher's exact test. Survival curves were estimated by the Kaplan‐Meier method. A *P*‐value <0.05 was considered statistically significant.

## 3 RESULTS

### 3.1 Baseline characteristics of patients

**Figure 1:**
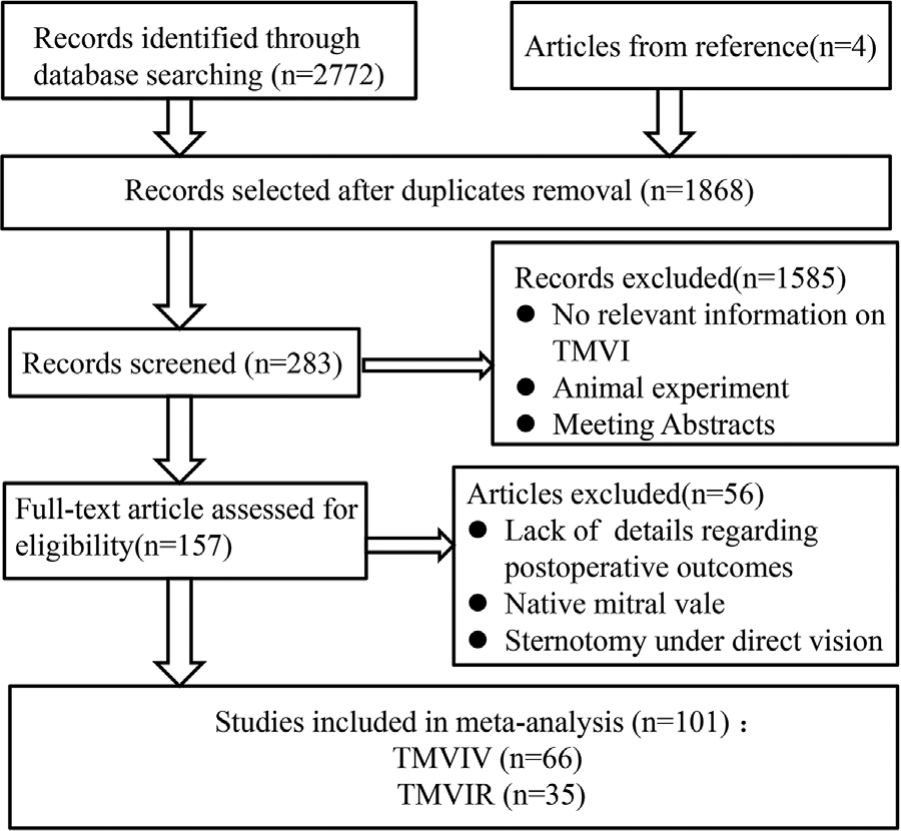
Flowchart of study selection process. TMVIR, transcatheter mitral valve‐in‐ting implantation; TMVIV, transcatheter mitral valve‐in‐valve implantation

From 2009 to March 31, 2018, 66 published reports^9^, ^10^, ^11^, ^12^, ^13^, ^14^, ^15^, ^16^, ^17^, ^18^, ^19^, ^20^, ^21^, ^22^, ^23^, ^24^, ^25^, ^26^, ^27^, ^28^, ^29^, ^30^, ^31^, ^32^, ^33^, ^34^, ^35^, ^36^, ^37^, ^38^, ^39^, ^40^, ^41^, ^42^, ^43^, ^44^, ^45^, ^46^, ^47^, ^48^, ^49^, ^50^, ^51^, ^52^, ^53^, ^54^, ^55^, ^56^, ^57^, ^58^, ^59^, ^60^, ^61^, ^62^, ^63^, ^64^, ^65^, ^66^, ^67^, ^68^, ^69^, ^70^, ^71^, ^72^, ^73^, ^74^ describing 172 patients undergoing TMVIV implantation for degenerated mitral bioprostheses and 35 articles describing 73 patients undergoing TMVIR implantation^22^, ^38^, ^45^, ^75^, ^76^, ^77^, ^78^, ^79^, ^80^, ^81^, ^82^, ^83^, ^84^, ^85^, ^86^, ^87^, ^88^, ^89^, ^90^, ^91^, ^92^, ^93^, ^94^, ^95^, ^96^, ^97^, ^98^, ^99^, ^100^, ^101^, ^102^, ^103^, ^104^, ^105^, ^106^ for failed annuloplasty rings were identified (Figure 1). The patients were diagnosed with MR, MS, or mixed lesions according to the articles but the failure modes of 34 patients were unknown. The characteristics of the studies included in this meta‐analysis are summarized in Table 1.

**Table 1:** Baseline characteristics of all patients, TMVIV and TMVIR

Clinical information	All patients	TMVIV	TMVIR
Total sample size, *n*	245	172	73
Age (years, mean ± SD)	73.0 ± 12.1 (169)	74.5 ± 12.5 (119)	70.0 ± 10.8 (60)
Male gender, %	50.6 (84/166)	46.5 (53/114)	59.6 (31/52)
Logistic EuroSCORE, %	19.1 ± 12.8 (91)	36.4 ± 17.1 (69)	37.8 ± 21.4 (22)
STS score, %	15.6 ± 13.5 (130)	16.8 ± 15.2 (86)	13.4 ± 9.0(44)
STS score >8%	70.8 (92/130)	72.1 (62/86)	68.2 (30/44)
Comorbidity, %	76.0 (114/150)	80.0 (80/100)	68.0 (34/50)
PH	35.2 (63/179)	37.7 (46/122)	29.8 (17/57)
CAD	15.2 (29/179)	14.8 (18/122)	19.3 (11/57)
CRF	33.0 (59/179)	35.2 (43/122)	28.1 (16/57)
Diabetes	16.2 (29/179)	17.2 (21/122)	14.0 (8/57)
AF	36.9 (66/179)	41.8 (51/122)	26.3 (15/57)
Other valve dysfunction, %	32.7 (49/150)	40.0 (40/100)	21.4 (9/50)
AR	4.4 (8/179)	6.6 (8/122)	0.0 (0/57)
AS	10.6 (19/179)	9.8 (12/122)	12.3 (7/57)
TR	19.0 (34/179)	23.0 (28/122)	10.5 (6/57)
TS	0.6 (1/179)	0.0 (0/122)	1.8 (1/57)
History of heart surgery, %	53.8 (86/160)	51.6 (63/122)	60.5 (23/38)
SAVR	20.0 (32/160)	20.5 (25/122)	18.4 (7/38)
CABG	27.5 (44/160)	27.0 (33/122)	28.9 (11/38)
TVR	13.8 (22/160)	13.9 (17/122)	13.2 (5/38)
Mitral valve failure mode, %			
MR	55.2(116/210)	49.3 (71/144)	68.2 (45/66)
MS	29.5 (62/210)	31.9 (46/144)	24.2 (16/66)
Mixed	15.3 (32/210)	18.8 (27/144)	7.6 (5/66)
NYHA ≥III, %	98.2(165/168)	97.3 (108/111)	100.0 (57/57)
LVEF (%, mean ± SD)	46.7 ± 14.1 (106)	51.2 ± 11.5 (73)	36.7 ± 14.5 (33)
MR severe or ≥Grade 3, %	69.4 (129/186)	63.3 (76/120)	80.3 (53/66)
Mean transmitral gradient (mmHg, mean ± SD)	12.1 ± 5.9 (155)	12.8 ± 5.9 (121)	9.5 ± 5.2 (34)

AF, atrial fibrillation; AR, aortic regurgitation; AS, aortic stenosis; CABG, coronary artery bypass grafting; CAD, coronary artery disease; CRF, chronic renal failure; LVEF, left ventricular ejection fraction; MR, mitral regurgitation; MS, mitral stenosis; NYHA, new york heart association; PH, pulmonary hypertension; SAVR, surgical aortic valve replacement; SD, standard deviation; STS, the Society of Thoracic Surgeons; TMVIR, transcatheter mitral valve‐in‐ring implantation; TMVIV, transcatheter mitral valve‐in‐valve implantation; TR, tricuspid regurgitation; TS, tricuspid stenosis; TVR, tricuspid valve replacement or repair.

### 3.2 Procedure

Transapical (TA) access was performed in 127 (55.2%) cases, and transseptal (TS) access (via a transfemoral or transjugular venous route) was performed in 91 (37.7%) patients. In addition to TA and TS access, a direct transatrial access using a sheath placed directly into the left atrium via a right anterior thoracotomy was also used in two patients.^67^, ^101^ The “TA + TS” access was utilized for the Melody valve.^36^, ^62^ Transcatheter valves were used in all patients and included the SAPIEN XT (*n* = 120, Edwards Lifesciences, Irvine, CA), SAPIEN (*n* = 47, Edwards Lifesciences), SAPIEN 3 (*n* = 26, Edwards Lifesciences), Melody (*n* = 18, Medtronic, Minneapolis, MN), Tiara (*n* = 4, Neovasc Inc, Richmond, Canada), Lotus (*n* = 3, Boston Scientific, Natick, MA), Tendyne (*n* = 1, Abbott, Abbott Park, IL), and Direct Flow Medical transcatheter valve system (DFM) (*n* = 9, Direct Flow Medical Inc, Santa Rosa, CA).

### 3.3 Clinical outcomes

Table 2 shows the in‐hospital outcomes. The MVARC technical success rate (assessed at exit from the catheterization laboratory) was 93.5%. Five technical failures occurred in the TMVIV group and 13 occurred in the TMVIR group. Fourteen patients (5.7%) died before discharge including two intraoperative (due to left ventricular apical perforation) and 12 postoperative deaths. Thirteen patients developed access‐site bleeding after TMVIV implantation. Other vascular complications occurred in two patients including one case of thrombosis on the ventricular aspect of the mitral valve prosthesis^28^ and one case of left ventricular (LV) apical pseudoaneurysm. Most patients (98.2%) were categorized as NYHA grade II or lower postprocedure. The mean transmitral gradient decreased after both procedures (*P* < 0.001), and the NYHA function improved significantly (Table 3). The cumulative events at 30 days and 6 months postoperatively are shown in Table 4. Three and nine additional deaths developed at 30 days and 6 months, respectively. Two additional pseudoaneurysms, two additional thromboses (one due to device failure: leaflet thickening and reduced leaflet motion), and two additional device migrations occurred, and two patients required an implantable cardiac defibrillator during the 30‐day follow‐up period. Two additional thromboses, two additional device migrations, and three additional device failures developed during the 6‐month follow‐up period.

**Table 2:** In‐hospital outcomes according to MVARC criteria

	All patients	TMVIV	TMVIR
Technical success, %	93.5 (229/245)	97.1 (167/172)	84.9 (62/73)
Death, %	5.7 (14/245)	5.2 (9/172)	6.8 (5/73)
Cardiovascular, %	4.1 (10/245)	2.9 (5/172)	6.8 (5/73)
Valve migration, %	2.9 (7/245)	2.3 (4/172)	4.1 (3/73)
LVOTO, %	1.6 (4/245)	0.0 (0/172)	5.5 (4/73)
Postprocedural MR^a^, %			
Trace/none	69.3 (147/212)	73.8 (107/145)	59.7 (40/67)
Mild or grade 1	23.1 (49/212)	20.7 (30/145)	28.3 (19/67)
>Mild	7.6 (16/212)	5.5 (8/145)	12.0 (8/67)
Access site and vascular complication, %			
Bleeding	6.1 (15/245)	8.7 (15^b^/172)	0.0 (0/73)
Thrombus	0.4 (1/236)	0.6 (1/163)	0.0 (0/73)
Pseudoaneurysm	0.4 (1/236)	0.0 (0/163)	1.4 (1/73)
Stroke, %	1.6 (4/245)	1.7 (3/172)	1.4 (1/73)
MI, %	0.0 (0/245)	0.0 (0/172)	0.0 (0/73)
New arrhythmia, %	2.0 (5/245)	1.7 (3/172)	2.7 (2/73)
Acute kidney injury, %	4.5 (11/245)	4.1 (7/172)	5.5 (4/73)
Postprocedural mean transmitral gradient, (mmHg, mean ± SD)	5.1 ± 2.5 (140)	5.1 ± 2.5 (96)	5.1 ± 2.5 (44)
NYHA (at latest follow‐up) ≤II, %	94.0 (109/116)	92.0 (69/75)	97.6 (40/41)

LVOTO, left ventricular outflow tract obstruction; MI, myocardiac infarction; MR, mitral regurgitation; MVARC, Mitral Valve Academic Research Consortium; NYHA, New York Heart Association; SD, standard deviation; TMVIR, transcatheter mitral valve‐in‐ring implantation; TMVIV, transcatheter mitral valve‐in‐valve implantation.

^a^ Including paravalvular leak and intervalvular regurgitation.

^b^ Including 2 left ventricular apical perforations in the procedure and 13 access‐site bleeding after the procedure.

**Table 3:** Mean transmitral gradient and NYHA before and after the procedure

	Mean transmitral gradient, (mmHg, mean ± SD)			NYHA ≥ III, %		
	Pre	post	*P*‐valve	Pre	post	*P*‐valve
All patients	12.1 ± 5.9 (155)	5.1 ± 2.5 (140)	<0.001	98.2 (165/168)	6.2 (7/113)	<0.001
TMVIV	12.8 ± 5.9 (121)	5.1 ± 2.5 (96)	<0.001	97.3 (108/111)	8.1 (6/74)	<0.001
TMVIR	9.5 ± 5.2 (34)	5.1 ± 2.5 (44)	<0.001	100.0 (57/57)	3.6 (1/39)	<0.001

NYHA, New York Heart Association; SD, standard deviation; TMVIR, transcatheter mitral valve‐in‐ring implantation; TMVIV, transcatheter mitral valve‐in‐valve implantation.

**Table 4:** Postprocedure cumulative events

	All patients		TMVIV		TMVIR	
	30‐day	6‐month	30‐day	6‐month	30‐day	6‐month
Death, %	8.1 (17/210)	23.4 (26/111)	7.5 (11/147)	18.8 (16/85)	9.5 (6/63)	38.5 (10/26)
Pseudoaneurysm, %	2.1 (3/142)	4.8 (3/63)	2.1 (2/95)	3.6 (2/55)	2.1 (1/47)	12.5 (1/8)
Stroke, %	2.8 (4/142)	6.3 (4/64)	3.2 (3/95)	5.4 (3/56)	2.1 (1/47)	12.5 (1/8)
MI, %	0.0 (0/142)	0.0 (0/60)	0.0 (0/95)	0.0 (0/53)	0.0 (0/47)	0.0 (0/7)
Thrombus, %	2.1 (3/142)	7.5 (5/67)	3.2 (3/95)	8.3 (5/60)	0.0 (0/47)	0.0 (0/7)
Device migration, %	4.9 (7/142)	13.0 (9/69)	5.3 (5/95)	11.7 (7/60)	4.3 (2/47)	22.2 (2/9)
Device failure, %	0.7 (1/142)	6.6 (4/61)	1.1 (1/95)	5.6 (3/54)	0.0 (0/47)	14.3 (1/7)
ICD, %	1.4 (2/142)	3.2 (2/62)	1.1 (1/95)	1.9 (1/54)	2.1 (1/47)	12.5 (1/8)
ASD closure, %	6.3 (9/142)	13.0 (9/69)	7.4 (7/95)	11.7 (7/60)	4.3 (2/47)	22.2 (2/9)

ASD, atrial septal defect; ICD, implantable cardiac defibrillator; LVOTO, left ventricular outflow tract obstruction; MI, myocardiac infarction; TMVIR, transcatheter mitral valve‐in‐ring implantation; TMVIV, transcatheter mitral valve‐in‐valve implantation.

### 3.4 Subgroup analysis

#### 3.4.1 Comparison of different mitral failure modes

**Figure 2:**
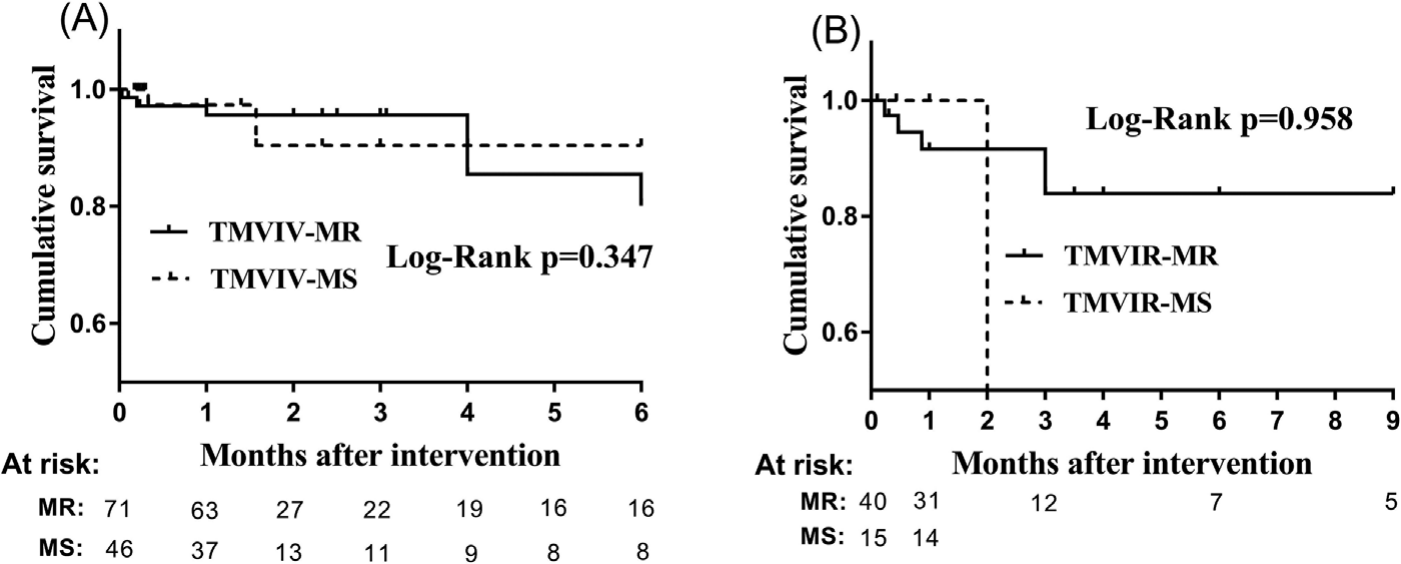
Kaplan‐Meier overall survival curves for patients with different mitral failure modes (MR and MS) in the TMVIV procedure (A) and the TMVIR procedure (B). MR, mitral regurgitation; MS, mitral stenosis; TMVIR, transcatheter mitral valve‐in‐ring implantation; TMVIV, transcatheter mitral valve‐in‐valve implantation

The patients with MR or MS in the TMVIV and TMVIR groups had similar baseline characteristics, except that the MR patients had a higher mean Logistic EuroSCORE and more previous heart surgeries (65.8% vs 37.2%, *P* = 0.017) in the TMVIV group and a higher percentage of the MR patients in the TMVIR group had an STS score >8% (75.0% vs 44.4%, respectively, *P* = 0.034). Regarding the clinical outcomes (Table 5), no significant differences were observed between the MR and MS groups, but MS patients in the TMVIR group had a higher mean transmitral gradient (*P* = 0.002). Different mitral failure modes (MR and MS) did not affect the patient's overall survival in both the TMVIV and TMVIR procedures (*P* = 0.347 and 0.958, respectively) (Figure 2).

**Table 5:** In‐hospital outcomes of different mitral failure modes in TMVIV and TMVIR

	TMVIV			TMVIR		
	MR	MS	*P*‐valve	MR	MS	*P*‐valve
Technical success, %	94.3 (50/53)	100.0 (35/35)	0.405	86.0 (37/43)	92.9 (13/14)	0.837
Death, %	7.7 (3/39)	0.0 (0/24)	0.404	6.7 (3/45)	0.0 (0/14)	>0.999
Valve migration, %	7.7 (3/39)	0.0 (0/24)	0.404	10.3 (3/29)	0.0 (0/10)	0.556
LVOTO, %	0.0 (0/39)	0.0 (0/24)	‐	6.9 (2/29)	10.0 (1/10)	>0.999
Postprocedural MR^a^, %						
None/trace	84.9 (45/53)	77.7 (23/30)	0.349	70.0 (28/40)	66.7 (10/15)	>0.999
Mild or grade 1	11.3 (6/53)	16.7 (5/30)	0.724	25.0 (10/40)	20.0 (3/15)	0.974
>Mild	3.8 (2/53)	6.8 (2/30)	0.954	5.0 (2/40)	13.3 (2/15)	0.853
Access site and vascular complication, %						
Bleeding	5.1 (2/39)	4.2 (1/24)	>0.999	0.0 (0/29)	0.0 (0/ 10)	‐
Thrombus	2.6 (1/39)	0.0 (0/24)	>0.999	0.0 (0/29)	0.0 (0/10)	‐
Pseudoaneurysm	0.0 (0/39)	0.0 (0/24)	‐	0.0 (0/29)	0.0 (0/10)	‐
Stroke, %	0.0 (0/39)	0.0 (0/24)	‐	0.0 (0/29)	0.0 (0/ 10)	‐
MI, %	0.0 (0/39)	0.0 (0/24)	‐	0.0 (0/29)	0.0 (0/ 10)	‐
New arrhythmia, %	5.1 (2/39)	0.0 (0/24)	0.521	3.4 (1/29)	0.0 (0/10)	>0.999
Acute kidney injury, %	12.8 (5/39)	4.2 (1/24)	0.487	3.4 (1/29)	10.0 (1/10)	0.452
Postprocedural mean transmitral gradient (mmHg, mean ± SD)	5.6 ± 2.7 (45)	5.0 ± 3.2 (28)	0.378	4.2 ± 1.9 (21)	6.7 ± 2.4 (15)	0.002
NYHA (at latest follow‐up) ≤II, %	94.3 (33/35)	100.0 (14/14)	>0.999	94.7 (18/19)	100.0 (9/9)	>0.999

LVOTO, left ventricular outflow tract obstruction; MI, myocardiac infarction; MR, mitral regurgitation; MS, mitral stenosis; NYHA, New York Heart Association; SD, standard deviation; TMVIR, transcatheter mitral valve‐in‐ring implantation; TMVIV, transcatheter mitral valve‐in‐valve implantation.

^a^ Including paravalvular leak and intervalvular regurgitation.

#### 3.4.2 Comparison of different access routes

**Figure 3:**
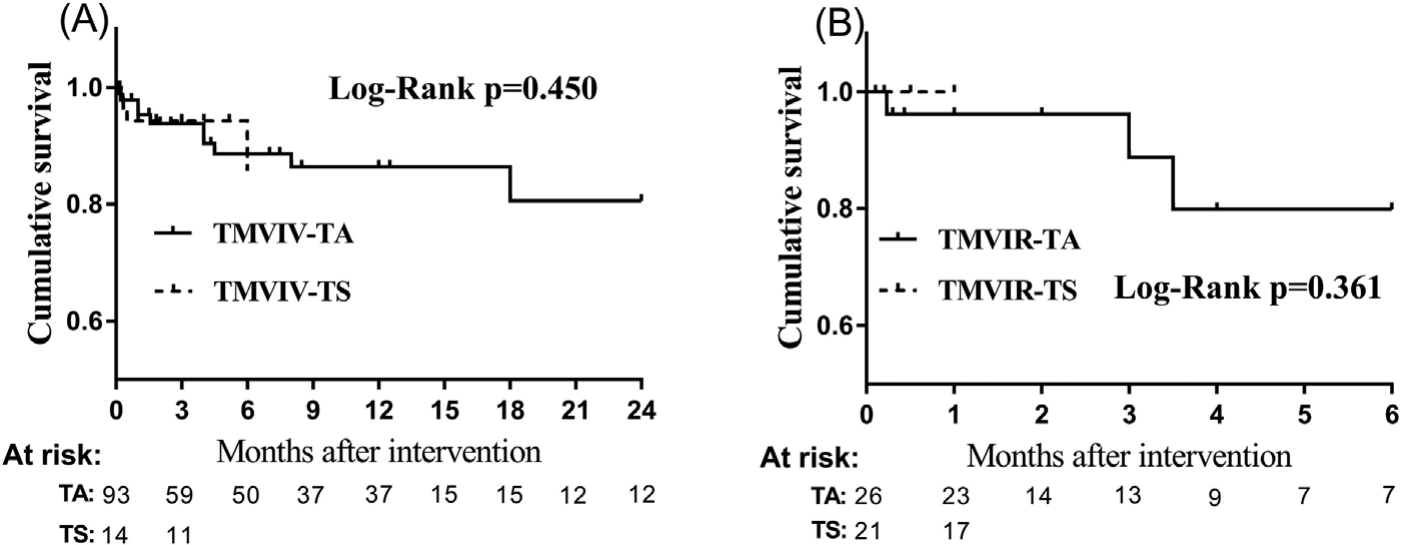
Kaplan‐Meier overall survival curves for patients with different access routes (TA and TS) in the TMVIV procedure (A) and the TMVIR procedure (B). TA, transapical; TMVIR, transcatheter mitral valve‐in‐ring implantation; TMVIV, transcatheter mitral valve‐in‐valve implantation; TS, transseptal

Patients who underwent the TMVIV procedure via TA access had a higher incidence of concomitant aortic or tricuspid valve dysfunction than those who underwent the TMVIV procedure via TS access (56.3% vs 16.7%, *P* = 0.001). More patients in the TA group had a previous surgical or interventional procedure (58.1% vs 34.6%, *P* = 0.035). Patients who underwent the TMVIR procedure via TA access had a higher mean logistic EuroSCORE, and MR was more severe in patients who underwent the procedure via TS access (93.3% vs 56.0%, *P* = 0.001). No significant differences were observed in clinical outcomes at discharge (Table 6). Different access routes (TA and TS) did not affect the patient's overall survival in both the TMVIV and TMVIR procedures (*P* = 0.450 and 0.361, respectively) (Figure 3).

**Table 6:** In‐hospital outcomes of different access routes

	TMVIV			TMVIR		
	TA	TS	*P*‐valve	TA	TS	*P*‐valve
Technical success, %	98.9 (93/94)	95.1 (58/61)	0.337	89.7 (35/39)	86.7 (26/30)	0.427
Death, %	3.2 (3/94)	6.6 (4/61)	0.555	10.3 (4/39)	3.3 (1/30)	0.528
Valve migration, %	1.1 (1/94)	1.6 (1/61)	>0.999	0.0 (0/39)	10.0 (3/30)	0.155
LVOTO, %	0.0 (0/94)	0.0 (0/61)	‐	5.1 (2/39)	6.7 (2/30)	>0.999
Postprocedural MR^a^, %						
Trace/none	98.9 (92/93)	100.0 (61/61)	>0.999	63.2 (24/38)	44.0 (11/25)	0.134
Mild or grade 1	1.1 (1/93)	0.0 (0/61)	>0.999	23.7 (9/38)	44.0 (11/25)	0.090
>Mild	0.0 (0/93)	0.0 (0/61)	‐	13.2 (5/38)	12.0 (3/25)	>0.999
Access site and vascular complication, %						
Bleeding	8.5 (8/94)	8.2 (5/61)	0.945	0.0 (0/39)	0.0 (0/30)	‐
Thrombus	1.1 (1/94)	0.0 (0/61)	>0.999	0.0 (0/39)	0.0 (0/30)	‐
Pseudoaneurysm	0.0 (0/94)	0.0 (0/61)	‐	0.0 (0/39)	3.3 (1/30)	0.435
Stroke, %	2.1 (2/94)	1.6 (1/61)	>0.999	2.6 (1/39)	0.0 (0/30)	>0.999
MI, %	0.0 (0/94)	0.0 (0/61)	‐	0.0 (0/39)	0.0 (0/30)	‐
New arrhythmia, %	3.2 (3/94)	0.0 (0/61)	0.417	2.6 (1/39)	0.0 (0/30)	>0.999
Acute kidney injury, %	8.5 (8/94)	3.3 (2/61)	0.337	7.7 (3/39)	0.0 (0/30)	0.327
Postprocedural mean transmitral gradient (mmHg ± SD)	5.1 ± 3.1 (39)	5.4 ± 2.5 (43)	0.652	4.3 ± 2.3 (19)	5.9 ± 2.6 (21)	0.071
NYHA (at latest follow‐up) ≤II, %	93.9 (46/49)	100.0 (12/12)	>0.999	100.0 (18/18)	93.3 (14/15)	0.455

LVOTO, left ventricular outflow tract obstruction; MI, myocardiac infarction; NYHA, New York Heart Association; SD, standard deviation; TA, transapical; TMVIR, transcatheter mitral valve‐in‐ring implantation; TMVIV, transcatheter mitral valve‐in‐valve implantation; TS, transseptal

^a^ including paravalvular leak and intervalvular regurgitation.

#### 3.4.3 Percentage of oversized valves

We collected manufacturer inner diameter (ID) measurements for MOSAIC and HANCOCK bioprostheses and the size of the ES valve (Table 7) and compared the mean % of oversized valves ([ES size‐Manufacture ID]/Manufacturer ID* 100%). We excluded those undersized valves used in MR and MS patients. The mean % of oversized valves was 6.79 ± 5.37% (*n* = 29, range 0–20.53%) in MR patients and 4.16 ± 3.46% (*n* = 11, range 0–8.33%) in MS patients, but the difference was not significant (*P* = 0.141).

**Table 7:** Size selection in MR and MS patients

Prior valve type	Prior valve size (mm)	Manufacture ID (mm)	Size of ES valve	No. of MR	No. of MS	No. of migration
MOSAIC	23	20.5	23	2	0	0
	25	22.5	23	2	3	0
	27	24	26	2	2	0
	27	24	29	1	0	0
	29	26	26	2	1	0
	29	26	29	4	0	0
	31	28	26	2	0	0
	31	28	29	0	1	0
	33	30	29	3	0	0
HANCOCK	25	22.5	23	1	1	0
	27	24	26	4	2	1
	29	26	26	4	1	0
	29	26	29	3	0	0
	31	28	29	4	0	0
	33	30	29	1	0	0

ES, Edward SAPIEN, SAPIEN XT, and SAPIEN 3 valve; ID, internal diameter; MR, mitral regurgitation; MS, mitral stenosis.

## 4 DISCUSSION

The TMVIV or TMVIR procedure for degenerated mitral bioprostheses or failed annuloplasty rings appears to be a feasible option for high‐risk, inoperable patients. With the development of these techniques, an increasing number of patients can obtain good clinical outcomes, but many technical issues such as size and access route selection remain undefined. No standard guidelines exist for the TMVIV and TMVIR procedures and no long‐term clinical trials (including randomized trials) have been performed to evaluate these techniques. Several ongoing trials have been designed to evaluate the safety and performance of some devices (Tiara (NCT03039855), Highlife (NCT02974881), Medtronic Intrepid (NCT03242642), Twelve (NCT02428010), and the Caisson (NCT02768402) and the effectiveness of the SAPIEN 3 (NCT03193801) valve for the TMVIV procedure, but no data have been reported to date. In our review of 245 patients using TMVIV or TMVIR, the technical success rate was 93.5% and the in‐hospital mortality was 5.8%. The transmitral gradient decreased and NYHA function improved significantly, with few postprocedural complications. TMVIV and TMVIR procedures are highly efficient and safe. However, in our analysis, the overall 6‐month mortality was 23.4% (18.5% and 38.5% for TMVIV and TMVIR, respectively). Long‐term follow‐up data were limited (only 45.3% patients completed 6 months of follow‐up). To date, few studies including at least 20 patients have reported long‐term (with at least 1 year) mortality, and those mortalities varied as follows: 16.9% (*n* = 248, 1 year),^107^ 9.6% (*n* = 38, more than 376 days),^63^ and 42.4% (*n* = 24, 3 years).^108^ Additionally, comparisons with surgical mitral valve replacement were unavailable, and only one ongoing controlled trial (NCT03242642) is currently being conducted.

Wunderlich et al^96^ found that the transcatheter valve could be adequately deployed within failed bioprostheses, but the failed annuloplasty ring was too oval‐shaped to adapt to the configuration of the implanted valve and would be more likely to develop MR. Notably, the TMVIV procedure was associated with a higher technical success rate (97.1%) than the TMVIR procedure (84.9%, *P* = 0.001), and a lower postprocedural MR rate (*P* = 0.039) was also observed for the TMVIV procedure. This result was similar to that of Yoon et al^107^ (248 patients from a transcatheter mitral valve replacement multicenter registry). Of the five patients (2.9%) experiencing TMVIV failures, two were due to operative error, and three others were due to prosthesis migration: two into the left atrium and one into the left ventricle. Regarding the TMVIR procedure, technical issues caused all the failures (*n* = 11, 15.1%). Three of the 11 failures were due to partial ring dehiscence following prosthesis deployment, and one failure was related to incomplete ring expansion. Anatomical differences also account for the differences in the technical success rate and post‐procedure MR rate between the TMVIV and TMVIR procedures. The native mitral valve leaflets in failed rings may disrupt the valve fixation and alter motion of the transcatheter mitral leaflets and the failed ring may be deformed during deployment.

### 4.1 MR versus MS failure mode

Different mitral failure modes are associated with specific anatomic and hemodynamic characteristics. Performing a retrograde implantation in the presence of MS is more difficult than performing this procedure in the presence of MR due to the difficulty in crossing the bioprostheses with the wire and implanting the transcatheter valves. Some issues arise when the TA access route is chosen. Pagnotta et al^33^ reported that they changed to the TS access route because they were unable to cross the degenerated mitral valve bioprostheses after multiple attempts. However, no clinical data exist to address these issues at present, and almost all on‐going trials are only designed for MR patients. In our analysis, both patients with MR or MS achieved a high technical success rate and good clinical outcomes, and no significant differences were observed in the early clinical outcomes for the TMVIV and TMVIR procedures. However, the TA access was used in most MR patients (57.3%) and the TS access was chosen for most MS patients (56.9%, *P* = 0.029). To determine whether the TA and TS access routes were associated with different outcomes in MS patients, we compared the outcomes between the TA (*n* = 22) and TS (*n* = 26) access routes, but no significant differences were found.

### 4.2 TA versus TS access

The first transcatheter mitral valve implantation in humans was performed via TS access.^3^ In 2013, all TMVIV procedures were successfully performed via a TA approach.^63^ The TA route was used for most procedures. The TA access has the following advantages: (1) direct and co‐axial access; (2) shorter distance; and (3) better control during deployment. The TA is also the first choice for patients with peripheral vascular disease. The TS route also has several advantages, including being less invasive and can be done under local anesthesia. However, the TS access route can cause an iatrogenic atrial septal defect (ASD), and some patients (16.5%) required an ASD occluder. A study by Frerker et al showed no significant differences in clinical outcomes, especially bleeding and vascular complications between the TA and TS access route (*P* = 0.35 and *P* = 0.13, respectively); however, TS access was associated with improved survival (*P* = 0.045).^108^ In our analysis, most patients (55.2%) were treated via TA access, and no differences in clinical outcomes and survival curves were observed, especially for bleeding and vascular complications, for both the TMVIV and TMVIR procedures. Similar baseline characteristics were shared by the two procedures.

### 4.3 Analysis of valve migration

Valve migration was the main (37.5%) cause of technical failure for these procedures. Eight patients developed valve migration into the LA either instantly in the cath lab or delayed/after exiting from the catheterization laboratory. In the TMVIV procedure, two patients (1.2%) developed valve migration to the LA after deployment, and five patients (8.3%) developed delayed migration. We found two cases (2.7%) of instant migration to the LA, one case (1.4%) of migration to the LV and no delayed migrations in the TMVIR procedure. Although no significant differences were observed between the TMVIV and TMVIR procedures for delayed migration (*P* = 0.452), more cases of migration were associated with the TMVIR procedure.

### 4.4 Optimal valve positions

The optimal positions for valves for both the MIVIV and TMVIR procedures have not been determined. The SAPIEN/SAPIEN XT device should be implanted in the mitral position with 10–20% of the device located atrially.^55^ In our analysis, four patients underwent successful TMVIV surgery using the SAPIEN/SAPIEN XT valve, and the positions were as follows: 1/3 of the valves were above the annular level and 2/3 beneath this level^34^; 30% of the new prostheses were on the atrial side, and the SAPIEN was 10% higher on the atrial end^27^ with 10% bias toward the atrial side.^40^ The 1‐year follow‐up results were reported in only one of the four cases. At the 6‐month follow‐up, one of the three pericardial leaflets was stuck in the closed position; however, the patient was in excellent clinical condition. Fluoroscopy showed an “hour‐glass” shape of the SAPIEN XT valve due to a final positioning that favored the atrial side (30% to 35% on the atrial side).^52^ Unlike the TMVIV procedure, no consensus exists regarding the optimal position for the TMVIR procedure. A total of nine articles reported variable positions. The three different positions of the ES valve are as follows: (1) less atrium more ventricle: 1/3 in the atrium and 2/3 in the ventricle^75^, ^101^, ^104^ or 40% in the atrium and 60% in the ventricle^90^, ^105^; (2) half above and half below the mitral ring^95^; (3) more atrium less ventricle: 40% ventricular and 60% atrial configuration.^92^ Two articles reported the position of the Melody valve as follows: 20% in the atrium and 80% in the ventricle^98^ and 40% in the atrium and 60% in the ventricle.^100^ All patients had successful TMVIR surgery and good clinical outcomes before discharge, and no migrations occurred. The position of the valve in the TMVIR procedure may be not as important as that in the TMVIV procedure. We found that the valve could be deployed more conically, and the position has little influence on valve expansion.

### 4.5 Sizing considerations

Currently, the ID of the valve set by the manufacturers is the most important criteria for transcatheter valve sizing for the TMVIV and TMVIR procedure. In a series by Cheung et al,^63^ the pre‐existing prosthesis was oversized by a minimum of 10% according to the manufacturer's ID. However, Seiffert et al^70^ suggested that oversizing should be limited in cases with a rigid xenograft stent because it may result in uneven stent expansion and leaflet distortion. In our study, the mean extent of oversizing in MR patients was greater than that in MS patients, but both mean proportions were less than 10%, with no significant difference between groups (*P* = 0.141). Due to a lack of data regarding migration, we could not determine whether migration occurred more frequently in patients with a larger mean extent of oversizing.

### 4.6 Limitations

This is an observational study and all patients’ data were obtained from published articles collected during a comprehensive and systematic search. Only a few articles reported long‐term follow‐up data; therefore, evaluation of long‐term outcomes was not possible. All studies included in this study lacked control groups.

## 5 CONCLUSION

Use of the TMVIV or TMVIR procedure for degenerated mitral bioprostheses or failed annuloplasty rings is a highly feasible, safe, and effective technique for the treatment of either valve stenosis or regurgitation for those patients who are not candidates for repeat surgery. Both the TMVIV and TMVIR procedures are associated with excellent short‐term clinical outcomes. The technical success rate of TMVIV was significantly higher than that for TMVIR, and the MR rate of TMVIV was significantly lower than that for TMVIR. No significant differences in short‐term outcomes were observed between the TA and TS access groups, especially regarding vascular complications. Technical criteria, such as size selection and valve location, have not been established for transcatheter mitral valve implantation. Larger clinical trials are required to determine the durability and long‐term outcomes of TMVIR and TMVIV.

## CONFLICTS OF INTEREST

There are no conflicts of interest to declare.
